# 

**Published:** 1903-10-31

**Authors:** 


Crown 8vo. Illustrated. Price **2s. 6d.** net.

**ANÆSTHETICS.** By J. BLUMFELD, M.D.Cantab., Senior Anæsthetist to St. George's Hospital; Anæsthetist to the Grosvenor Hospital for Women and Children, London, and to the National Dental and Metropolitan Hospitals.

*Lancet.*—"....In writing this handbook the author has been quite successful." *Australasian Medical Gazette.*—"We can strongly commend this book." *Medical Chronicle.*—"The book is one which we can heartily recommend to senior students and practitioners."

London: BAILLIÈRE, TINDALL & COX, 8 Henrietta St., Covent Garden.

**THE HANDBOOK OF THE OPEN-AIR TREATMENT**

By Dr. CHARLES REINHARDT

(Visiting Physician to Hailey Sanatorium, Wallingford, and Stourfield Park Sanatorium, Bournemouth),

Contains Views, Descriptions, and Information respecting 30 Sanatoria in Great Britain.

Price: Cloth, 2/6; Paper, 1/- (postage 4d.)

The "HEALTH RESORT" Publishing Office, 379 Strand, London, W.C.

JUST ISSUED. EIGHTH EDITION. Crown 8vo. 10s. 6d.

**PHARMACY, MATERIA MEDICA AND THERAPEUTICS**

Is now rewritten and printed in large type, and is made a companion to the following volume. It contains an account of the action **of every drug in the B.P.**, as well as a section upon all the newest drugs, with an Introduction to the Art of Dispensing. With woodcuts, prescriptions, &c.

Also FOURTH EDITION. 1055 pages, 8vo. 16s.

**DICTIONARY OF TREATMENT.**

Containing a Revised and up-to-date Article upon every Diseased Condition, whether Medical, Surgical, Gynæcological, or Ophthalmological, as well as Articles upon Poisoning, Drowning, and the various Surgical Emergencies and Accidents, arranged for Rapid Reference.

By W. WHITLA, M.A., M.D., Professor of Materia Medica, Queen's College, Belfast; Senior Physician to the Royal Victoria Hospital, &c.

The Publisher will supply both volumes, carriage paid, for 21/-

London: HENRY RENSHAW, 356 Strand.

WORKS BY Mr. McADAM ECCLES, M.S., F.R.C.S., Assistant Surgeon St. Bartholomew's Hospital, &c.

**HERNIA.**

SECOND EDITION, 1902. Price 7/6 net. Illustrated by Numerous Photographs.

**THE IMPERFECTLY DESCENDED TESTIS.**

Just published. Profusely Illustrated. Price 7/6.

London: BAILLIÈRE, TINDALL & COX.

### NOTICE TO CORRESPONDENTS.

All MSS., Letters, Books for Review, and other matters intended for the Editor should be addressed The Editor, "The Hospital," The Hospital Buildings, 28 & 29 Southampton Street, Strand, W.C.

The Editor cannot undertake to return rejected MS., even when accompanied by stamped directed envelope.

**Notice to Advertisers and Subscribers.**

All Advertisements, Orders for Copies of The Hospital, and Business Communications should be addressed to **THE MANAGER (Not to the Editor),** The Hospital, 28 & 29 Southampton Street, Strand, London, W.C.

The Cost of Subscription, post free, is as follows:—Per week, 3½d.; per quarter, 3s. 9d.; per half-year, 7s. 6d.; per annum, 15s. Foreign and Colonial:—Per annum, 19s. 6d., payable, in all cases, in advance.

**NOTICE.—Vol. XXXIV. of "The Hospital" (April 1903 to September 1903), in handsome cloth binding, will shortly be on sale at the office of this Journal, price 7s. 6d. Cases for binding the Numbers contained in this Volume 1s. 6d. Index to Vol. XXXIV., 2½d., post free.**

**The Monthly part for November will be ready on November 2. Price 1s., by post, 1s. 3d.**

### THE STUDENT OF MEDICINE.

### APPOINTMENTS.

**James J. F. Bourke**, L.R.C.P.&S.Irel., Medical Officer at Hughenden, Queensland. **Walter P. Cockle**, B.A., M.D., B.Ch., B.A.O., L.M.Dubl., Medical Officer and Public Vaccinator for the Borough of Ealing and the Parish of Twyford Abbey. **E. Evans**, M.R.C.S., L.R.C.P.Lond., Medical Officer of the Holbeach Union Workhouse. **Lucinda Forster**, M.B., B.S.Lond., Medical Officer, Quarantine Office, Suez. **Alex. Frew**, M.B.,Ch.B. Edin., Medical Officer to Witwatersrand Deep, Driefontein Deep, and Knight's Central Gold Mines, near Johannesburg, Transvaal. **Norman Glegg**, M.B., Ch.B.Edin., Assistant Medical Officer to the Birmingham Parish Infirmary. **Wm. Henry Harbison**, L.R.C.P.&S. Edin., Health Officer for Wallaroo, South Australia. **J. E. Hewlett**, M.B., C.M., Clinical Assistant to the Chelsea Hospital for Women. **Edward C. Hope**, M.R.C.S.Eng., Medical Officer at Winton, Queensland. **Lancelot M. Hungerford**, L.R.C.P.&S. Irel., Health Officer at Busselton, West Australia. **G. Jameson Johnston**, M.A., M.B., F.R.C.S.I., Honorary Surgeon, Masonic Orphan Boys' School, Dublin. **Sydney H. Long**, M.D.Cantab., Medical Officer of Health to Cromer. **Charles Mackey**, M.B., Ch.B., Junior Resident Medical Officer to the Manchester Children's Hospital, Pendlebury. **Clive Riviere**, M.D.Lond., M.R.C.P., Physician to the Out-patients at the City of London Hospital for Diseases of the Chest, Victoria Park. **William Francis Roe**, L.R.C.P.I. and L.R.C.S.I. and L.M., Physician to the Holloway and North Islington Dispensary. **J. Clark Wilson**, M.D.Edin., Clinical Assistant to the Samaritan Free Hospital.

Westminster Hospital Medical School.—The following appointments have recently been made to vacancies in Lectureships at the School:—Lecturer in Pathology: **R. Grainger Hebb**, M.D. Lecturer in Physiology: **B. Louis Abrahams**, M.B. Lecturer in Biology: **A. Campbell Stark**, B.Sc.

The Best and Most Authoritative Book on Nursing.

### GENERAL NURSING.

By EVA C. E. LÜCKES.

(Matron of the London Hospital.)

Fourth Edition now Ready. Price **5/-**

### THE SERMON IN THE HOSPITAL

(from "The Disciples").

By Mrs. HAMILTON KING.

Miniature Edition. Leather **1/-** net.

### HOME NURSING

For Young Housekeepers with Special Reference to

**THE CARE OF INFANTS AND CHILDREN.**

BY

ELIZABETH J. MOFFETT, B.Sc., M.D.Lond.

Illustrated, 107 pages, **1/6.**

KEGAN PAUL, TRENCH, TRÜBNER & CO.,

Dryden House, 43 Gerrard Street, W.

### Hospital Matrons,

Do you know the best book on how to provide for the Hospital Household? In

### HOSPITAL EXPENDITURE: The Commissariat,

Price **2/6** post free, you will find every question considered which bears upon this important subject. The varying circumstances of large and small institutions are carefully considered, and from the making of beef-tea to the complete cost of diets everything that can interest and assist an intelligent manager is within its pages.

You can compare your methods with those of other Hospitals, because careful comparisons are made, even in the matter of the sale of waste material. Matrons about to occupy new posts will find this book invaluable, as it will place them at once in that position of power which knowledge gives.

### The Cottage Hospital Matron will find useful

### COTTAGE HOSPITALS,

**By Sir HENRY BURDETT, K.C.B.**

Price **10/6** post free, 3rd Edition, and a little pamphlet on the

### FURNISHING AND APPLIANCES OF A COTTAGE HOSPITAL,

**By the Hon. SYDNEY HOLLAND,**

Price **6d.** post free; and those matrons who have no secretarial assistance, and all secretaries and medical officers will find invaluable

### THE COTTAGE HOSPITAL CASE-BOOK,

**Or, REGISTER OF PATIENTS.**

Price **10/-** post free.

It is arranged for 150 in-patient cases, with rulings for 8 weeks' payments to each case.

Larger size, containing space for about 300 in-patient cases, **12/6** post free.

All these books can be obtained at all Booksellers', and at
THE SCIENTIFIC PRESS, 28 Southampton Street, Strand, W.C.

### WORKS FOR NURSES.

**LESSONS ON MASSAGE.** By Mrs. MARGARET D. PALMER, Manager of the Massage Department and Instructor of Massage to the Nursing Staff of the London Hospital. Second Edition enlarged and revised; pp. xvi. + 262 with 118 Illustrations, plain and coloured. *Just Published.* Price 7/6 net.

**A MANUAL FOR STUDENTS OF MASSAGE.** By Miss M. A. ELLISON, L.O.S. With 50 original Illustrations. Price 3/6 net.

**A MANUAL FOR HOME NURSING.** By BERNARD MYERS, M.D., M.R.C.S., Surgeon and Lecturer to St. John's Ambulance Association. *Just Published.* Price 2/6 net.

**NOTES ON HOME NURSING.** By Miss D. M. GOLDIE, Lecturer on Nursing, Hygiene, &c., to the County Councils of London, Surrey and Middlesex. *Just Published.* Price 1/6 net.

**THE FEEDING AND HYGIENE OF INFANTS AND CHILDREN.** By JOHN McCAW, M.D., M.R.C.P., Physician to the Belfast Hospital for Children. *Just Published.* Price 2/6.

**DISEASES OF THE HAIR, INCLUDING GREYNESS, BALDNESS, &c., AND THEIR TREATMENT.** By DAVID WALSH, M.D., Western Hospital for Diseases of the Skin. Price 2/6 net.

**MENSTRUATION AND ITS DISORDERS.** By ARTHUR E. GILES M.D., B.Sc., F.R.C.S., Surgeon Chelsea Hospital for Women. Price 2/6 net.

**MOVABLE ATLAS OF THE ANATOMY AND PHYSIOLOGY OF THE FEMALE GENERATIVE ORGANS AND OF PREGNANCY.** Second Edition. With Text. By ARTHUR E. GILES, M.D., F.R.C.S. Price 3/- net.

**MOVABLE ATLAS OF THE ANATOMY AND PHYSIOLOGY OF THE CHILD.** With Explanatory Text. By D'ARCY POWER, M.A.Oxon, M.B., F.R.C.S. Price 3/- net.

**BAILLIÈRE'S POPULAR MOVABLE ATLAS OF THE ANATOMY AND PHYSIOLOGY OF MAN.** Size 16 by 7½ inches. With Explanatory Text. By W. S. FURNEAUX, Special Science Teacher London School Board. Price 3/6 net.

London: BAILLIÈRE, TINDALL & COX, 8 Henrietta Street, Covent Garden.

NOW READY,

A REPRINT OF

A NURSING HANDBOOK

TOGETHER WITH S. MAW, SON AND SONS'

COMPLETE CATALOGUE OF NURSING REQUISITES.

The above work is now republished at a cost of 1s. per copy. Messrs. S. M., S. and S. have been compelled to make this charge on account of the extreme popularity of the work and the great expense incurred in its production.

*Post Free on receipt of 1s.*

Nurses' Bags.

S. MAW, SON and SONS,

7 to 12 ALDERSGATE STREET, LONDON, E.C.

